# EXploring Patterns of Use and Effects of Adult Day Programs to Improve Trajectories of Continuing Care (EXPEDITE): Protocol for a Retrospective Cohort Study

**DOI:** 10.2196/60896

**Published:** 2024-08-30

**Authors:** Matthias Hoben, Colleen J Maxwell, Andrea Ubell, Malcolm B Doupe, Zahra Goodarzi, Saleema Allana, Ron Beleno, Whitney Berta, Jennifer Bethell, Tamara Daly, Liane Ginsburg, Atiqur SM - Rahman, Hung Nguyen, Kaitlyn Tate, Kimberlyn McGrail

**Affiliations:** 1 School of Health Policy and Management Faculty of Health York University Toronto, ON Canada; 2 Faculty of Nursing College of Health Sciences University of Alberta Edmonton, AB Canada; 3 School of Pharmacy University of Waterloo Waterloo, ON Canada; 4 ICES Toronto, ON Canada; 5 Alzheimer Society of York Region Aurora, ON Canada; 6 Member of the Advisory Committee, Helen Carswell Chair in Dementia Care Faculty of Health York University Toronto, ON Canada; 7 Max Rady Faculty of Health Sciences College of Medicine University of Manitoba Winnipeg, MB Canada; 8 Manitoba Centre for Health Policy Faculty of Health Sciences University of Manitoba Winnipeg, MB Canada; 9 Centre for Care Research Western Norway University of Applied Sciences Winnipeg, MB Canada; 10 Cumming School of Medicine University of Calgary Calgary, AB Canada; 11 Arthur Labatt Family School of Nursing Faculty of Health Sciences Western University London, ON Canada; 12 Institute of Health Policy, Management and Evaluation Dalla Lana School of Public Health University of Toronto Toronto, ON Canada; 13 KITE Research Institute Toronto Rehabilitation Institute University Health Network Toronto, ON Canada; 14 Centre for Health Services and Policy Research School of Population and Public Health The University of British Columbia, BC Canada

**Keywords:** adult day care centers, aged, program evaluation, cohort studies, routinely collected health data

## Abstract

**Background:**

Adult day programs provide critical supports to older adults and their family or friend caregivers. High-quality care in the community for as long as possible and minimizing facility-based continuing care are key priorities of older adults, their caregivers, and health care systems. While most older adults in need of care live in the community, about 10% of newly admitted care home residents have relatively low care needs that could be met in the community with the right supports. However, research on the effects of day programs is inconsistent. The methodological quality of studies is poor, and we especially lack robust, longitudinal research.

**Objective:**

Our research objectives are to (1) compare patterns of day program use (including nonuse) by province (Alberta, British Columbia, and Manitoba) and time; (2) compare characteristics of older adults by day program use pattern (including nonuse), province, and time; and (3) assess effects of day programs on attendees, compared with a propensity score–matched cohort of older nonattendees in the community.

**Methods:**

In this population-based retrospective cohort study, we will use clinical and health administrative data of older adults (65+ years of age) who received publicly funded continuing care in the community in the Canadian provinces of Alberta, British Columbia, and Manitoba between January 1, 2012, and December 31, 2024. We will compare patterns of day program use between provinces and assess changes over time. We will then compare characteristics of older adults (eg, age, sex, physical or cognitive disability, area-based deprivation indices, and caregiver availability or distress) by pattern of day program use or nonuse, province, and time. Finally, we will create a propensity score–matched comparison group of older adults in the community, who have not attended a day program. Using time-to-event models and general estimating equations, we will assess whether day program attendees compared with nonattendees enter care homes later; use emergency, acute, or primary care less frequently; experience less cognitive and physical decline; and have better mental health.

**Results:**

This will be a 3-year study (July 1, 2024, to June 30, 2027). We received ethics approvals from the relevant ethics boards. Starting on July 1, 2024, we will work with the 3 provincial health systems on data access and linkage, and we expect data analyses to start in early 2025.

**Conclusions:**

This study will generate robust Canadian evidence on the question whether day programs have positive, negative, or no effects on various older adult and caregiver outcomes. This will be a prerequisite to improving the quality of care provided to older adults in day programs, ultimately improving the quality of life of older adults and their caregivers.

**Trial Registration:**

ClinicalTrials.gov NCT06440447; https://clinicaltrials.gov/study/NCT06440447

**International Registered Report Identifier (IRRID):**

PRR1-10.2196/60896

## Introduction

Across the globe, societies are struggling to meet the needs of an aging population [1-5]. The increasing prevalence of dementia [6-8] and comorbid chronic conditions [9,10] lead to complex care needs [9,10] and to greater family or friend caregiver burden [11-14] (ie, “the extent to which caregivers perceive that caregiving has had an adverse effect on their emotional, social, financial, physical, and spiritual functioning” [15]). In response, health systems provide a range of ongoing care and supports to older adults and their caregivers—in Canada commonly referred to as continuing care [2,16]. Continuing care can be provided in an older adult’s private home, in the community (eg, an adult day program), or in a variety of congregate care settings including independent living, retirement homes, supportive or assisted living, or nursing homes (NHs) [17,18]. Governments have identified NHs as a major driver of public continuing care costs [17,19-21]. To mitigate pressures on public continuing care systems, and to meet aging in place preferences of older adults and their caregivers [22-24], reforms have implemented aging in place strategies. These strategies largely include (1) reserving NH care to those with the most complex care needs, and (2) improving access to an array of publicly funded continuing care options in the community [2].

Adult day programming is such a continuing care option to support aging in place [25-32]. Older adults in need of continuing care usually attend these programs for parts of the day, returning to their homes overnight (but overnight services are provided by some day programs). As this literature illustrates [25-32], the number of days a person attends a day program can vary widely, depending on the program and health jurisdiction, from a couple of days or month to daily attendance. The amount of time an individual attends also varies from a few hours or day to all day, or sometimes during nights, and so do admission criteria, supports and services offered, and funding models.

Despite these variations, day programs have unique characteristics that set them apart from other continuing care options. Day programs employ care staff and admit people with a certain level of support needs [30,33]. This distinguishes them from senior or community centers [34] and creative arts programs [35], which are open to independent older adults, do not employ care staff, and are organized more informally. Unlike home care [36] or in-home respite [37], day programs serve groups of older adults in a setting external to the attendee’s home [30,33], supporting social interactions and caregiver respite [32]. Unlike geriatric day hospitals, which provide medical, therapeutic, and rehabilitative care for a few weeks [38], day programs prioritize social and recreational activities, and they do so for long term (often for months or years) [30,33]. Day program services and supports usually include transportation; meals; recreational activities (eg, playing games, musical activities, crafting, and painting); socializing with other clients and day program staff; physical, cognitive, and spiritual activities; social work counseling; and case management support. Personal, nursing, and medical care are often not provided, or only to a limited extent, depending on the program and health system.

Recent literature reviews [28-32,39] reveal a growing body of evidence that suggests that day program attendance may be associated with attendees’ improved mental health, cognition, loneliness, quality of life, perceived health, physical functioning, use of polypharmacy, and mortality. These reviews also suggest that attendance may be associated with older adults’ delayed admissions to congregate care, reduced risk for hospitalization, improved caregiver burden, and caregivers’ feelings of competence, mental health, and well-being. However, reviews point to inconsistent findings, methodological limitations, and substantial heterogeneity of included studies. For example, a Canadian 1-group pre-post study suggested that Geriatric Depression Scale scores decreased (fewer depressive symptoms) from 5.0 at admission to a day program to 3.3 at discharge (*P*=.007). A quasi-experimental study comparing depressive symptoms between day program attendees with dementia and nonattendees with dementia in the United States [40] found no group differences. However, on days of attendance, the proportion of caregivers who reported depressive symptoms for attendees decreased over time (from 32/133, 24% to 25/133, 19%; *P*<.02). A Canadian randomized controlled trial [41] found no difference in depressive symptoms between day program attendees and wait-listed nonattendees.

Across the literature, four key knowledge gaps persist: (1) we generally know little about the characteristics of day program attendees and nonattendees, or about those with different patterns of use. (2) We lack longitudinal data on changes in the aforementioned outcomes. (3) Generally, the methodological quality of available studies is poor [32], and we lack robust, large-scale, longitudinal evidence of older adult day programs on day program attendees—especially those living with dementia. With few notable exceptions [42,43], we especially lack current research on Canadian day programs with most research originating from the US or Canadian studies often dating back several decades [25,26,44]. (4) Differential effects of day programs on persons with multiple, intersecting vulnerabilities are poorly understood, despite inequity concerns [39,45-47]. Advanced age puts individuals at risk of ageism; physical and cognitive disabilities may expose them to ableism; the majority of older adults and their caregivers are women, often experiencing gender inequities; and giving and receiving care are associated with substantial health care costs, disproportionally affecting those with low income [48]. Racism or transphobia or homophobia can further increase these pressures, severely affecting older adults and their caregivers [49,50].

Our study will address these knowledge gaps comprehensively, rigorously, and simultaneously. We will address the following 3 research objectives:

1. Explore patterns of day program use (eg, variations in time to first attendance, monthly hours of attendance, ongoing versus interrupted attendance, and total time of day program exposure), using latent class analyses (LCA), and compare the frequency of each latent use class between provinces and over time.
2. Compare older adults’ demographic, social, and health characteristics (eg, age, sex, physical or cognitive disability, area-based deprivation indices, and caregiver availability or distress) by day program use or nonuse class, province, and time.
3. Assess whether, compared with a propensity score–matched control group of nonattendees, day program attendees enter care homes at later points in time; use emergency, acute, or primary care less frequently; experience less cognitive and physical decline; have better mental health; and have less distressed caregivers. We will assess potential modification of these effects by day program use or nonuse class, age, sex, and social determinants of health (eg, area-based deprivation indices).

## Methods

### Study Design

Using an integrated knowledge translation (iKT) approach [51,52], we partnered with a cross-Canadian team of experts to design this population-based retrospective cohort study (ClinicalTrials.gov; NCT06440447) covering the Canadian provinces of Alberta, British Columbia, and Manitoba, and we will collaborate with our experts throughout the study. Experts include older adults (some with dementia), their caregivers, Alzheimer societies, caregiver organizations, day program staff and managers, and government and health system decision makers. They will provide intimate knowledge of day programs, and the experience of attending them or caring for an attendee, which will help us interpret and contextualize our findings. We will use deidentified clinical and health administrative data from each of the 3 provinces. Our study will follow the STROBE (Strengthening the Reporting of Observational Studies in Epidemiology) [53], and the REporting of studies Conducted using Observational Routinely-collected health Data (RECORD) [54] guidelines. Provincial data policies require data to remain in each respective province, preventing linkage across provinces and analyses of all data in one place. Therefore, in-house data analysts with each provincial health system will carry out the analyses separately with shared protocols and programs.

### Setting and Sample

Our study settings are community-based continuing care systems. Each province provides access to a range of publicly funded community-based continuing care services, including adult day programs [55-63]. Each provincial health system determines and enacts access criteria and provides services (directly or via contracted providers) [55-63]. Day program eligibility is assessed in each province, using comparable processes, criteria, and assessments (ie, the Resident Assessment Instrument—Home Care [RAI-HC], a standardized, valid, reliable assessment tool [64]) [60,65,66]. To be eligible, attendees need to have not only some care dependency but also the ability to cope to some extent with activities of daily living, ambulate or transfer with no or minimal assistance, be continent or independent in managing continence products, exhibit no or easily manageable responsive behaviors, and either be alone for extended periods or have a caregiver who requires respite. Our study cohort will include all individuals aged 65 years or older with an initial RAI-HC assessment completed between January 1, 2012, and December 31, 2021. We will follow everyone until they move into a care home, are lost to follow-up (eg, because of death, moving out of province, loss of public insurance eligibility), or until December 31, 2024 (the end of the period covered by our data). That will allow for a care trajectory of at least 3 years (for those with an initial RAI-HC assessment in December 2021), enabling us to assess the number and characteristics of individuals with different day program use patterns, and compare them with those who were never exposed to a day program.

### Sample Size Calculation

The yearly average number of completed RAI-HC assessments is ~20,000-30,000 in Alberta, ~34,000-39,000 in British Columbia, and ~10,000 in Manitoba [67]. About 50% of those assessed receive a reassessment within 12 months and another 10%-30% receive a reassessment after >15 months [68]. There are 89 publicly subsidized day programs in Alberta (~3300 spaces per day), 95 in British Columbia (~1500 spaces per day), and 70 in Manitoba (~1000 spaces per day) for a total of 254-day programs with 5800 spaces on any given day. Some day program users do not attend daily, but only 1 or a few days per week, so the number of unique attendees exceeds the number of spaces per day. This corresponds to >20,000 attendees per year (>200,000 within the study period), each with multiple assessments. Our study sample size will be large enough to detect small effects sizes. With Cox proportional hazards models, adjusted for covariates explaining an assumed 25% of effect variance (a=.05; power=0.8) [69], we require a total sample of 1327 participants to detect a hazard ratio for admissions to care homes of 0.6 (as can be expected based on a similar Canadian study [42]) in favor of day program attendees. Similarly, Kelly [43] was able to detect significantly fewer emergency department visits and hospital admissions per day among 812 day program attendees compared with 812 propensity score matched to nonattendees. Our expected sample size will be considerably larger than for those previous studies, allowing for complex statistical modeling.

### Data Sources

For each individual in our cohort, designated provincial health system analysts will link all records available within the study time frame from the following databases: (1) regional continuing care registries, documenting when an individual starts or stops receiving any community-based continuing care, including day programs, how these services change over time, and when an individual is admitted to a care home. (2) Population registries for each participant’s demographic data. (3) RAI-HC assessments [64], completed annually for people receiving long-term home care (60+ days), and to determine day program eligibility. The RAI-HC will provide data on older adults’ medical conditions, functional dependence, pain, cognitive impairment, mood, and behavioral problems. It also includes information on a person’s marital or partnership status, caregiver availability, whether that caregiver lives with the older adult, and caregiver distress. Additional caregiver characteristics are not included in the available provincial databases, posing a limitation to our quantitative analyses. However, a related prospective cohort study that we are conducting in Ontario will allow us to link comprehensive caregiver and older adult data, and we are currently conducting additional qualitative research that will illuminate how caregiver characteristics may affect day program use and outcomes. (4) Discharge abstract database (DAD) for information on all inpatient hospital stays, including diagnoses and length of stay. (5) National Ambulatory Care Report System (NACRS) for all emergency department visits and diagnoses. In British Columbia, we will use the physician payment file in addition, since NACRS is not collected in all emergency departments [70]. (6) Pharmaceutical information on outpatient prescription medications filled through a community pharmacy and covered by provincial drug formulary. (7) Care provider claims data for health service claims submitted for payment by health care providers (eg, general practitioners, nurse practitioners, geriatricians, geriatric psychiatrists, neurologists, therapists) to obtain information on general and specialist health services used by participants.

### Study Variables

#### Exposure

Our exposure will be different patterns of day program use or nonuse, based on information from the provincial continuing care registries, documenting the dates a person starts or stops attending a day program, days of attendance, and the duration of each visit. Day program use patterns will be determined, using LCAs (see *Statistical Analyses* section) [71]. We will categorize three continuous variables as low, low-moderate, high-moderate, or high use, using sample distribution quartiles: (1) time between first RAI-HC assessment and first attendance of a day program; (2) average number of hours of day program attendance (ie, total number of hours spent in a day program divided by the number of times attended); and (3) total number of days a person attended a day program. LCAs will also include a categorical variable, indicating whether a person consistently attended a day program or whether there were longer periods (several weeks) of nonattendance. Nonuse will be defined as no day program exposure at any time during a person’s continuing care trajectory.

#### Study Outcomes

The data sources noted above enable us to examine a range of important study outcomes. Data on the time between a person’s first RAI-HC assessment and admission to a care home will come from provincial continuing care registries. Symptoms of depression will be assessed using the validated RAI-HC Depression Rating Scale [72], with scores ranging from 0 to 14 and a cut point of 3 or higher representing clinically meaningful depressive symptoms [72,73]. We will capture physical and cognitive decline, using validated RAI-HC scales [64]: the Activities of Daily Living Hierarchy (ADLh) Scale [74] and the Cognitive Performance Scale (CPS) [75]. Both scales range from 0 (no impairment) to 6 (maximum impairment), and our outcomes will be dichotomous, indicating any increase (vs no change or a decrease) between the previous and follow-up measurement in each of these scales. Using care practitioner claims data, we will generate rates of different types of primary and specialist care use (eg, family physician, specialists, nursing practitioner, and allied health providers). We will use the DAD and NACRS databases to generate rates of emergency department registrations, hospital admissions, and days in hospital (including alternative level of care) [43]. Rates will be stratified by day program use or nonuse pattern.

#### Demographic, Social, and Health Characteristics

These will include older adults’ age, sex, marital or partnership status (population registries and RAI-HC), physical disability (ADLh Scale score of >3), and cognitive impairment (CPS score of >3). Available data sets include only a binary variable on biological sex (male or female) and no nonbinary information on gender identity. We will also include RAI-HC measures of caregiver availability (item G1e) and burden (items G2a-c). Finally, we will include 4 publicly available area-level measures from the Canadian Index of Multiple Deprivation [76,77]: residential instability (eg, housing insecurity, overcrowding, and frequent moves), economic dependency (high number of older adults, children younger than 15 years, and persons receiving government transfers), ethnocultural composition (eg, immigrants and racialized individuals), and situational vulnerability (eg, indigenous peoples, dwellings needing major repairs, and low education). Using Statistics Canada data, each measure is derived for 54,775 geographical dissemination areas, using 17 variables. Quintile-based ranks for each of the indices (1=least deprived to 5=most deprived) will be assigned to individuals based on their home’s postal code [77].

#### Propensity Score–Matching Variables

To compare outcomes between day program attendees and nonattendees, we will use propensity score matching [78] (for details, see Statistical Analyses section). Propensity scores aim to ensure a similar distribution of baseline variables among treatment (day program attendees) and control (nonattendees)—akin to what random assignment aims to accomplish in randomized trials [78]. Since we lack evidence on differences between day program attendees and nonattendees, our objective 2 analyses will be key to informing the selection of the exact covariates that will form the propensity score. We will derive covariates for day program attendees from the RAI-HC day program eligibility assessment (index date). For each day program attendee, we will identify potential matches as nonattendees whose first RAI-HC assessment was completed within ±3 months of the attendee’s index date (ie, admission to long-term home care at about the same time). This RAI-HC assessment will provide the relevant covariates to enable propensity score matching with day program attendees as of their index date.

Our first set of matching covariates will be RAI-HC variables used by health systems to determine day program eligibility [60,65,66]: physical functioning (ADLh Scale), cognition (CPS), behavioral symptoms (Aggressive Behavior Scale [79]), bladder or bowel continence (items I1, I3), availability of a caregiver (item G1e), and caregiver distress (items G2a-c). This will ensure that control participants are potentially eligible to a day program. Possible reasons for nonattendance include the lack of day program spaces, preference not to attend, inability to afford the required copayments, or not receiving a day program referral. Our experts assure us that the pool of potential matches far exceeds that of attendees, supporting the feasibility of this study and underscoring the lack of day program spaces. This approach excludes individuals whose care needs are either too low or too severe for day program eligibility, but it minimizes confounding by the matched variables and ensures comparable groups at baseline [80-82]. Finally, we will include a second set of matching covariates: health and social characteristics identified in objective 2 by which attendees and potentially eligible nonattendees differ and that overlap sufficiently between attendees and nonattendees (eg, age, sex, type or duration of publicly funded community care received before the matching index date, and deprivation indices).

#### Additional Covariates

Additional covariates for model adjustment will come from RAI-HC, DAD, NACRS, pharmaceutical, and claims records (eg, geriatric syndromes, medical diagnoses, and prescribed medications). We might also adjust for additional community care services (eg, in-home respite and home care).

### Statistical Analyses

#### Objective 1: Explore Patterns of Day Program Use

Using our day program cohort, we will conduct LCAs to determine the number of different day program use patterns, using the 4 variables described in the exposures section. LCAs are widely used to identify subgroups by clusters of characteristics (ie, parameters of day program use) [71]. In collaboration with our experts and guided by relevant literature, we will prespecify the expected number of classes. We will carry out LCAs separately in each province. We will run models with the prespecified number of classes, and with 1, 2, and 3 more and fewer classes than the number prespecified [71]. We will compare the fit between models, using bootstrap likelihood ratio tests [71], and select a final model that reflects the same number and types of classes in each province, balancing theoretical, conceptual, and statistical considerations. To assess temporal changes in the number of day program attendees within each use pattern, and differences between provinces, we will report and graphically plot the proportion (95% CI) of individuals within each latent class by quarter and province.

#### Objective 2: Compare Older Adults’ Characteristics by Day Program Use, Province, and Time

Using our full cohort of day program attendees and nonattendees, we will descriptively assess the distribution of sample characteristics over time and by province. In each province and quarter, we will report and plot graphically the proportion (95% CI) of individuals with each characteristic, stratified by day program use class versus nonuse. Using general estimating equations (GEEs) [83], we will assess whether the number of persons with each characteristic has changed over time and whether characteristics are associated with older adults’ day program use or nonuse pattern. We will run a separate GEE model for each characteristic within each province, with the respective characteristic as individual-level outcome. We will run binary logistic regressions for dichotomous variables (eg, sex) and ordinal regressions for categorical variables (eg, residential instability quintile). Models will account for repeated measures within individuals and include the independent variables year of assessment (to assess change in social determinants over time), use or nonuse class (to assess differences in social determinants by day program use), and an interaction between year and use or nonuse (to assess how social determinants differed between use and nonuse patterns by year). Using random-effects mixed regression models, we will pool provincial effects statistically. Other Pan-Canadian studies, such as the Canadian Network for Observational Drug Effect Studies [84], have successfully applied this approach and developed rigorous protocols to minimize bias and maximize consistency of regional analyses.

#### Objective 3: Assess Effects of Day Programs

To create a propensity score, we will run a logistic regression for each province with day program attendance or nonattendance as the dependent variable and adding matching covariates. We will use one-to-one matching (1 matched nonattendee for every attendee) [78]. We will use matching without replacement [85] and apply an optimal caliper matching algorithm [86]. As per best practice recommendations [87], we will use a caliper width of 0.2 of the SD of the propensity score’s logit. If this matching approach does not allow us to achieve a sufficient sample size, we will use propensity score quintiles for matching.

We will compare sample characteristics and study outcomes between attendees and nonattendees in every year and province, using bivariate statistical tests (eg, chi-square test or Fisher exact test for categorical variables, *t* tests, or ANOVAs for continuous variables, and their nonparametric equivalents if variables violate statistical assumptions). To assess the effect of day program exposure on time to care home admission, we will specify a multilevel time-to-event model with a health region–level random effect [88]. Health systems in each of the 3 provinces are divided into 5 health regions [89-91], and regional policies may cause clustering effects that our models must account for. Each model will include day program use or nonuse class as independent variable and will be adjusted for time-varying variables. These will include matching variables, if appropriate (ie, in case of group differences in matching variables over time or due to missing data) [80-82] and, if needed, additional covariates (eg, demographics, social determinants, medical or functional conditions, and non–day program community care). Covariates that differ between attendees and nonattendees with a *P* value of ≤.15 in the bivariate analyses will be considered for inclusion. We will add covariates stepwise, one-by-one, and remove those that cause collinearity issues or decrease model fit. As in objective 2, we will pool provincial effects statistically, using random-effects mixed regression models.

Using GEEs and a similar approach as for the time-to-event models (including separate models in each province and statistical pooling of their effects), we will assess whether the other study outcomes differ by day program use or nonuse pattern. Models will include each study outcome of interest as a dependent variable, day program use or nonuse class and time of assessment as independent variables, and similar covariates (using the same stepwise approach) as the time-to-event models. Models will also include a random term to account for repeated measures within individuals. The choice of a link function will be informed by the nature of the variable and theoretical and empirical considerations. For example, the number of hospital, emergency department, or physician visits has been shown to follow a zero-inflated negative binomial distribution, sometimes requiring an offset for the natural logarithm of person-time [92]. For continuous outcomes (eg, days spent in hospitals), we will use an identity link function, and for dichotomous outcomes (eg, presence or absence of depressive symptoms), we will use a logit link function. All models will apply multiple imputation in case of missing data, which we expect to be small based on our previous work with the administrative health care data sources used in this study.

### Ethical Considerations

We received ethics approvals from the York University Ethics Review Board, Human Participants Review Sub-Committee (e2022-412, December 1, 2022), the University of Alberta Health Research Ethics Board—Health Panel (Pro00127850, February 3, 2023), and the University of British Columbia Research Ethics Board (H24-01435, August 1, 2024), and we are in the process of obtaining ethics approval from the University of Manitoba Health Research Ethics Board.

## Results

Funded by an endowed research chair, the Helen Carswell Chair in Dementia Care (July 1, 2022, to June 30, 2027), this will be a 3-year study (July 1, 2024, to June 30, 2027). Starting on July 1, 2024, we will work with the 3 provincial health systems on data access and linkage, and we expect data analyses to start in early 2025.

## Discussion

### Principal Findings

Older adults, caregivers, and health systems urgently need solutions to empower older adults to receive care at home for longer [93]. There are few feasible solutions that target both, the older adult in need of care and their family or friend caregiver, but day programs are one of them [30,33]. Despite the knowledge that day programs could fill an immense and costly care gap [27-33,37,39,45-47,94,95], we lack the research needed to inform policy and drive practice change to make day programs more available [30,33]. This study will generate robust Canadian knowledge on whether day programs have positive, negative, or no effects on outcomes that matter most to older adults, their caregivers, and health systems. For example, day programs aim to support older adults and their caregivers to avoid or delay care home admissions; reduce or avoid costly and unnecessary emergency, acute, or primary care use; and improve the health and well-being of older adults and their caregivers [27-33,37,39,45-47,94,95]. However, the international research is inconclusive on whether or not day programs are effective in accomplishing these aims [27-33,37,39,45-47,94,95], and we especially lack robust, longitudinal, and cross-provincial Canadian research [25,26,44]. Therefore, this study will provide critical knowledge that is urgently needed by health systems. First, we will determine how many persons are attending day programs in the 3 participating Canadian provinces, what their patterns of use look like, whether these patterns have changed over time, and similarities and differences of these patterns between provinces. Second, we will assess how characteristics of older adults who attend day programs differ from those who do not attend day programs. Finally, we will assess whether day programs are effective in delaying admissions to care homes, reducing emergency, acute and primary care, and in improving various outcomes related to older adults’ health and well-being.

Our iKT approach, in which we have been closely partnering with older adults (some with dementia), their caregivers, Alzheimer societies, caregiver organizations, day program staff and managers, and government and health system decision makers, will ensure that our research addresses issues that these groups have deemed a priority. It will further facilitate rapid translation of these findings into policy and practice changes. Results will be disseminated in a variety of ways. Staying true to our iKT approach, we will invite, encourage, and empower our experts to participate in, coauthor, or lead these activities (to the extent our experts wish to be involved and have capacity to do so).

### Limitations

While this study has various important strengths, including the use of comprehensive, population-based, cross-provincial health administrative data, and application of robust statistical methods, there are some limitations. First, important variables, such as older adult quality of life, various social determinants of health, or day program characteristics, are not available in the administrative health care data available to us. Second, the health administrative data used in this study do not allow for identification of caregiver health administrative data, preventing linkage of caregiver and older adult data. Therefore, our team currently also carries out a prospective cohort study (ClinicalTrials.gov; NCT06496945) in which we will collect these missing variables to fill the mentioned gaps. Finally, quantitative data may suggest the presence or absence of an effect, but they may be more limited in explaining the mechanisms leading up to the effect or the reasons of the lack of an effect. Our program of research includes a realist literature review and comprehensive qualitative work to address the lived experience of older adults and their caregivers in day programs and the “how and why” of day program effects (or the lack thereof).

In webinars in years 2 and 3, researchers, trainees, and experts will copresent key research findings on specific topics, such as effects of day programs in general (ie, on individuals with dementia, caregivers, and health systems); variation of day program effects in various equity-deserving groups or by day program characteristics; or jurisdictional differences in day program structures, policies, characteristics, and effects. Thirty to 50 additional experts (not members of our advisory committee) will be invited to participate per webinar, including provincial or regional health system policy makers, Alzheimer Societies, caregiver organizations, day program operators or managers or staff, individuals with dementia, and caregivers. In particular, webinars will offer the opportunity for discussion about relevance of findings within and across jurisdictions and for cross-provincial learning (learning health systems).

Researcher, trainee, and expert team members will also codevelop a series of briefing documents that highlight key messages of our research. Documents will target health system policy makers and day program operators and managers. They will be a valuable tool to support desired directions and action post project funding. In year 2, we will hold a series of workshops to engage experts in a facilitated, deliberative process of developing alternative approaches that improve day program effects on individuals with dementia, their caregivers, and health systems.

We are planning the preparation of several peer-reviewed manuscripts (cocreated by researchers, trainees, and experts). Publications might include (among others) (1) this research protocol of our study; (2) a manuscript comparing the number and characteristics of day program attendees over time and across participating regions; (3) a comparison of older adults by day program use or nonuse stratified by health region; and (4) several papers (~3-5) on the effects of day programs on older adults. Team members will give presentations at conferences within Canada (eg, Canadian Association for Health Services and Policy Research, Ontario Long Term Care Association, National Health Leadership Conference, Canadian Alliance for Long Term Care, Canadian Association on Gerontology, and Congress of the Humanities and Social Sciences) and internationally (Gerontological Society of America, International Association of Gerontology and Geriatrics). Experts will be invited to participate in symposia, copresent, or lead presentations.

In year 3, key messages will be used to develop various lay summaries and an easily accessible, animated summary project video that can also be used for educational training on aging in place and care of individuals with dementia and their caregivers in the community. These will be posted on our study website and on our team members websites.

In conclusion, this study will identify essential elements of day programs and how they can be improved. We will provide critical evidence for health systems to help them leverage the full potential of day programs to provide appropriate care, prevent inequities, and mitigate the need for emergency, hospital, and congregate care. Ultimately, we will improve the quality of life of older adults (including those with dementia) and their caregivers, alleviate caregiver burden, and reduce social costs associated with poor health and well-being. Future studies will expand this research to additional health jurisdictions.

## Abbreviations

ADLh: Activities of Daily Living Hierarchy Scale
CPS: Cognitive Performance Scale
DAD: discharge abstract database
GEE: general estimating equation
iKT: integrated knowledge translation
LCA: latent class analysis
NACRS: National Ambulatory Care Report System
NH: nursing home
RAI-HC: Resident Assessment Instrument—Home Care
RECORD: REporting of studies Conducted using Observational Routinely-collected health Data
STROBE: Strengthening the Reporting of Observational Studies in Epidemiology

## Data Availability

This study uses population-based clinical and administrative health care data, routinely collected, and owned by the participating health regions. Provincial data policies do not allow for public sharing or access of these data. Data are not allowed to be removed from regional repositories. Guided by the study team, data in each region will be analyzed by health data analysts employed by the respective health care system. Study findings will then be pooled across health regions, using shared protocols and statistical codes. Access may be granted to those who meet prespecified criteria for access, available at on the websites of (1) the Alberta SPOR SUPPORT Unit (AbSPORU) [96] (email: absporu@albertainnovates.ca), (2) Population Data BC [97] (email: dataaccess@popdata.bc.ca), and (3) Shared Health Inc [98] (email: SHResearch@sharedhealthmb.ca).
